# Potential role of resveratrol in abdominal cocoon syndrome: targeting inflammation, oxidative stress, and fibrosis via food-derived bioactive compounds resveratrol for treatment of abdominal cocoon syndrome

**DOI:** 10.3389/fphar.2026.1974332

**Published:** 2026-09-29

**Authors:** Hailong Guo, Minglei Bi

**Affiliations:** 1 Department of General Surgery, Jiading District Central Hospital Affiliated Shanghai University of Medicine & Health Sciences, Shanghai, China; 2 Shanghai Key Laboratory of Cancer System Regulation and Clinical Translation, Jiading District Central Hospital, Renji Hospital Jiading Branch, Shanghai, China; 3 Department of General Surgery I (Gastrointestinal Surgery), Shanghai Municipal Hospital of Traditional Chinese Medicine, Shanghai, China

**Keywords:** abdominal cocoon syndrome, drug delivery, fibrosis, inflammation, oxidative stress, resveratrol

## Abstract

Abdominal cocoon syndrome (ACS) is a rare refractory peritoneal fibrotic disease marked by intestinal encapsulation by dense fibrous membranes. Its pathogenesis involves chronic inflammation, oxidative stress, pathological angiogenesis, immune dysregulation and peritoneal fibrosis, and targeted pharmacotherapy remains unavailable. Resveratrol, a natural medicine-food homologous polyphenol, exerts anti-inflammatory, antioxidant, anti-angiogenic, immunomodulatory and anti-fibrotic effects through NF-κB, Nrf2, TGF-β/Smad and SIRT1 pathways, showing potential as an ACS adjuvant agent. Nevertheless, poor water solubility, heavy first pass metabolism, low bioavailability, photoisomerization and biphasic dose-dependent effects greatly hinder its clinical translation. Nanocarriers, prodrugs, stimulus-responsive carriers and local intraperitoneal delivery can ameliorate these pharmacokinetic defects. Direct evidence for resveratrol against ACS is still lacking. Further studies should establish standardized ACS animal models, clarify intraperitoneal pharmacokinetics and unify raw material quality standards. Resveratrol represents a promising candidate for ACS therapy with advances in precision delivery.

## 1 Introduction

ACS, also called sclerosing peritonitis, or encapsulating peritoneal sclerosis, is a rare bowel disorder, which was first reported by Foo et al. (1978). The main manifestation of ACS is that a part of or all intestine is wrapped by a layer of dense, tough, gray white, hard, and thick fibrous membrane. The ACS patients have clinical symptoms such as abdominal pain, abdominal distension, cessation of flatus and defecation, vomiting, and visible abdominal masses, etc. and some patients have no obvious clinical symptoms (Pokharel et al., 2019).

The whole incidence rate of ACS is unknown because of its rarity and heterogeneity of etiologies. According to published reviews, China, India and Turkey have a relatively high incidence rate of ACS. The mean age of ACS patients ranges from 7 years old to 87 years old, and the male is more prone to suffer from ACS than the female (Danford et al., 2018). Until now, the etiology of ACS has not been clarified clearly. The pathogenesis of ACS may be related to the congenital factors, retrograde menstruation, abdominal chronic inflammation, some diseases (cirrhosis, uremia, endometriosis, gynecologic neoplasms, and dermoid cyst rupture, etc.), and some medical operations such as peritoneal dialysis as well as organ transplantation (Danford et al., 2018), (Bahar et al., 2014).

Resveratrol (3,4,5-trihydroxystilbene) exemplifies the food-medicine convergence strategy for ACS. This natural phytoalexin is richly present in dietary sources: grapes (providing 0.24–14.3 mg/L in red wine), peanuts (0.02–1.79 mg/g), berries, and Polygonum cusp datum roots (Faisal et al., 2024). Its traditional use in Asian medicine and modern dietary supplement status (EFSA-recommended dose: ≤150 mg/day) underscores its dual functionality. Resveratrol exemplifies a classic “medicine-food homology' compound, where its dual roles as both a therapeutic agent and a dietary component bridge traditional medicine and modern nutrition. However, despite its promising pharmacological potential, resveratrol faces significant pharmacokinetic challenges, particularly its poor water solubility (approximately 0.03 mg/mL in aqueous solutions) and the resultant low oral bioavailability in humans (<1%) (Faisal et al., 2024). These limitations substantially restrict its clinical translation and therapeutic efficacy. In the following sections, we will emphasize innovative strategies to overcome these physicochemical barriers, including novel formulation approaches and delivery systems designed to enhance resveratrol’s solubility, stability, and ultimately, its biological activity in abdominal cocoon syndrome management.

This is a narrative review rather than a systematic review. Literature was retrieved from PubMed and Web of Science from database inception to July 2026. The main search terms included abdominal cocoon syndrome/encapsulating peritoneal sclerosis/sclerosing peritonitis, resveratrol, fibrosis, inflammation, oxidative stress, angiogenesis and immune regulation. Supplementary searches were performed for nutraceuticals, drug delivery and peritoneal fibrosis. Peer-reviewed full-text original articles and reviews were screened. Publications focusing on the pathological mechanisms of abdominal cocoon syndrome and multi-target pharmacological effects of resveratrol were prioritized, while duplicates, non-peer-reviewed abstracts, low-quality case reports and methodologically unreliable studies were excluded.

## 2 Overviews of resveratrol

Resveratrol was first isolated from *Veratrum album* in 1940, and until 2021, resveratrol can be discovered in more than 70 species of plants such as peanut, red wine, grape, mulberry, veratrum grandiflorum, semen cassia as well as polygonum cusp datum, etc (Faisal et al., 2024).

There are two isomers of resveratrol: *Trans*-resveratrol and *cis*-resveratrol. After the exposures of sunlight and ultraviolet radiation, *trans*-resveratrol can be converted to *cis*-resveratrol (Mota-Lugo et al., 2021). Two preparation methods can be applied to produce resveratrol: natural plants extraction method and synthesis method. Because of the high cost, natural plants extraction method has been rarely used. Currently, chemical synthesis, biological synthesis, and genetic engineering synthesis have been widely applied to produce resveratrol. However, similar to many kinds of TCM, resveratrol has the poor water solubility so that it is difficult to increase its bioavailability *in vivo*. Therefore, more and more researches have focused on the developments of various pharmaceutical preparations of resveratrol to solve this problem (Ha et al., 2021).

In 2003, Harvard University professor David Sinclair and his team stimulated a wave of researching on the biological effects of resveratrol (Howitz et al., 2003). Many studies have proved that resveratrol can extend the lifespans of both non-mammal animals and mammal animals (Bauer et al., 2004; Rascón et al., 2012; Baur et al., 2006; Pearson et al., 2008). Otherwise, many studies have reported that resveratrol possesses the anticancer effects on breast cancer, lung cancer, liver cancer, colon cancer, rectal cancer, gastric cancer, pancreatic cancer, prostatic cancer, cervical cancer, ovary cancer, skin cancer, leukemia, thyroid cancer, as well as osteosarcoma (Liu et al., 2018; Peng and Jiang, 2018; Li et al., 2019; Bian et al., 2020; Buhrmann et al., 2020; Dariya et al., 2020; Khusbu et al., 2020; Sun et al., 2020; Vargas et al., 2020; Wang et al., 2020; Yang et al., 2021; Lin et al., 2021; Ersöz and Adan, 2022).

Additionally, researchers also found that resveratrol had neuroprotective effects, regulating blood sugar effects, protecting blood vessels effects, and protecting liver effects, etc (Hui et al., 2018; Broderick et al., 2020; Labban et al., 2021; Sarroca et al., 2021; de Moraes et al., 2021). And these effects of resveratrol still need more researches to be proven further.

As mentioned above, finding a type of medicine which processes anti-inflammatory effect, anti-oxidative stress effect, regulating angiogenesis, anti-fibrosis effect, as well as immunoregulation simultaneously is of a great significance for the treatment of ACS. And resveratrol can meet these requirements, which will be discussed thoroughly below (Figure 1, Available: http://www.chemspider.com/ImagesHandler.ashx?id=392875&w=250&h=250).

**FIGURE 1:**
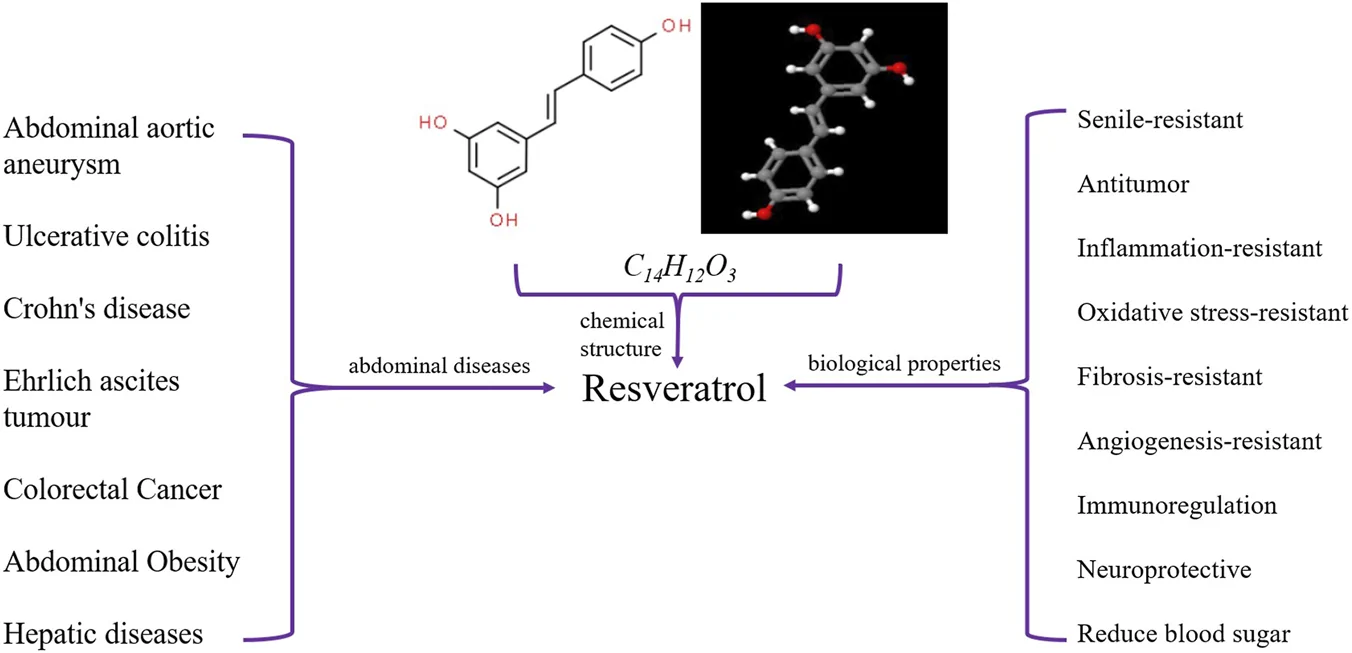
Chemical structure, biological properties and medicable abdominal diseases of resveratrol.

Therefore, as a kind of TCM, resveratrol has many pharmacological effects of which the inner mechanisms have been clarified thoroughly. It is promising that applying resveratrol will be a significant adjuvant therapy for ACS.

## 3 Overviews of ACS

ACS can be divided into primary ACS and secondary ACS. As one of the most rare and tough abdominal diseases, ACS is characterized by its secretiveness, diagnostic and therapeutic difficulty, poor prognosis, as well as high incidence rate of complications, which really impairs the patients’ life qualities and costs a huge number of medical resources. However, the pathogenesis of ACS is complicated and it is still unclear.

It has been reported that the following pathophysiological processes such as excessive inflammation, oxidative stress, angiogenesis, fibrosis, and immune dysregulation, contribute to the pathogenesis and development of ACS greatly. Inflammation-related causes of ACS are usually from the following inflammatory diseases such as chronic intestinal inflammation, chronic peritonitis, retroperitoneal inflammation, gynecological infections, abdominal tuberculosis, hepatitis, and nephritis, etc (Pan et al., 2024). Otherwise, abdominal surgeries and foreign bodies are also important contributors. Oxidative stress also plays an important role in the mechanisms of ACS. Gotloib et al. (2005) reported that sustained oxidative injury could cause the peritoneal sclerosis mainly by the loss of the mesothelial monolayer of peritoneum (Gotloib et al., 2005). Oxidative stress can lead to fibrosis by activating fibroblasts. Li C. et al. (2023) reported that the depletion of glutathione peroxidase-3 (GPX3) could orchestrate an oxidatively stressed extracellular microenvironment, which could lead to interstitial fibroblast activation and proliferation by upregulating protein kinase C-α (PKCα)/mitogen-activated protein kinase (MAPK)/signal transducer and activator of transcription 3 (STAT3) signaling pathway (Li C. et al., 2023).

Furthermore, angiogenesis has also been reported to have a close association with the pathogenesis of ACS. In 1963, Turner-warwick et al. found primarily in autopsy of lung tissue in patients with idiopathic pulmonary fibrosis (IPF) that the angiogenesis often occurred in pulmonary tissue of IPF, and the sites of angiogenesis were basically consistent with the sites of fibrosis (Turner-Warwick, 1963).

Fibrosis is the portent of sclerosis and its main pathological changes of fibrosis are an increase in fibrous connective tissue and a decrease in parenchyma cells within organ tissues. Minor fibrosis can be reversed by receiving proper treatments and major fibrosis will be transformed to sclerosis, which is irreversible and impairs the functions of affected organs. Intestinal fibrosis and peritoneal fibrosis are both causes and manifestations of ACS. Thereby, inhibiting intestinal fibrosis and peritoneal fibrosis should be emphasized greatly and this therapeutic thought is of great significance for the treatments of ACS. Moreover, it also has been reported that the pathogenesis and development of ACS are associated with immunity, and immunosuppression therapy has been considered as one of the most effective treatments of ACS. Taken together, how to treat ACS is a serious clinical problem due to its unknown and complex oncogenesis. To ease its huge economic and medical burdens, finding a type of proper medicine to treat ACS is urgently needed (Table 1).

**TABLE 1:** Pathophysiological processes and mechanisms in ACS.

Pathophysiological factor	Mechanisms	Clinical/Pathological manifestations	References
Inflammation	Chronic intestinal inflammation; Chronic peritonitis Retroperitoneal inflammation; Gynecological infections Abdominal TB; Hepatitis; Nephritis Abdominal surgery and foreign bodies	Persistent abdominal inflammation; Progression to fibrosis and sclerosis	(Pan et al., 2024)
Oxidative stress	Sustained oxidative injury→loss of peritoneal mesothelial monolayer→peritoneal sclerosis Depletion of GPX3 → oxidative microenvironment → fibroblast activation and proliferation via PKCα/MAPK/STAT3 pathway	Peritoneal fibrosis; Sclerosis; Tissue remodeling	(Gotloib et al., 2005); (Li C et al., 2023)
Angiogenesis	Angiogenesis often occurs in fibrotic tissue Sites of angiogenesis correspond with fibrosis (observed in IPF lung tissue)	Aberrant neovascularization associated with fibrotic progression	(Turner-Warwick, 1963)
Immune dysregulation	Abnormal immune responses contribute to ACS development; Immunosuppression can ameliorate progression	Potential benefit of immunosuppressive therapy in ACS	(Chen et al., 2022); (Xing et al., 2022)
Fibrosis	Increased fibrous connective tissue Decreased parenchymal cells Minor fibrosis reversible Major fibrosis → irreversible sclerosis	Intestinal fibrosis; Peritoneal fibrosis; Loss of organ functions	(Song et al., 2024); (Sun and Zheng, 2022)

Abbreviations: ACS: Abdominal cocoon syndrome; GPX3: Glutathione Peroxidase-3; IPF: Idiopathic pulmonary fibrosis; MAPK: Mitogen-Activated Protein Kinase; PKCα: Protein Kinase C-α; STAT3: Signal Transducer and Activator of Transcription 3; TB: Tuberculosis.

## 4 Beneficial effects and possible mechanisms of resveratrol on ACS

As mentioned above, excessive inflammation, oxidative stress, angiogenesis, fibrosis, as well as immunoregulation are main causes of ACS. As a type of TCM with a long history, resveratrol has been investigated deeply in its biological effects such as anti-inflammatory effect, anti-oxidative stress effect, regulating angiogenesis, anti-fibrosis effect, as well as immunoregulation in recent years (Gokce et al., 2017; Huang et al., 2019; Ding et al., 2022; Zhu et al., 2022) (Table 2; Figure 2).

**TABLE 2:** Summary of the multifaceted therapeutic mechanisms of resveratrol against core pathological processes in ACS.

Core pathological process in ACS	Protective effects of resveratrol	Molecular mechanisms & targets	Key supporting evidence (model)	Evidence strength	References
Chronic inflammation	Suppresses pro-inflammatory response; Alleviates chronic inflammation	- Inhibits NF-κB signaling pathway - Inhibits TGF-β/Smad signaling pathway - Downregulates TNF-α, IL-6, IL-1β, IL-8, IFN-γ - Upregulates anti-inflammatory IL-10 - Activates SIRT1/PGC-1α	PCOS rat model IBD, colitis Colon cancer models COPD rat model Clinical trials in diabetes, COPD, AD.	Animal models (non-ACS models) Level B	(Furat et al., 2018; Yang et al., 2020; Cai et al., 2020; Xu et al., 2016; Wang et al., 2017; Liu et al., 2014)
Oxidative stress	Reduces oxidative damage Enhances antioxidant defense	- Activates Nrf2/HO-1 antioxidant signaling pathway - Increases SOD activity; decreases MDA level - Reduces ROS production.- Improves GSH/GSSG ratio	AAA models IBD models UC patients Colon cancer models	*In vitro* cellular and animal models (non-ACS models) Level B	(Bilen et al., 2021; Kaneko et al., 2011; Palmieri et al., 2011; Hu Y. et al., 2019; Blagov et al., 2023; Samsamikor et al., 2016; Temi et al., 2014; Chiou et al., 2011; Zheng et al., 2019)
Pathological angiogenesis	Inhibits abnormal blood vessel formations	- Downregulates VEGF and MMPs (e.g., MMP-9) - Inhibits NF-κB and Sirt1 in endothelial cells - Suppresses CXCL-12/IL-6 signaling - Modulates MAPK signaling pathway	HUVEC *in vitro* Chick CAM model Osteoarthritis model Colorectal and breast cancer models	*In vitro* cellular and animal models (non-ACS models) Level B	(Uvez et al., 2020; Annabi et al., 2012; Pradhan et al., 2023; Khan et al., 2010; Yang et al., 2018; Xiong et al., 2021; Hu W. et al., 2019; Varghese et al., 2020)
Immune dysregulation	Modulates immune balance Restores immune homeostasis	- Modulates Th17/Treg cell balance - Reduces CRP and other inflammatory markers - Inhibits glycolysis-lactate axis to suppress Treg function - Regulates antitumor immunity	Colorectal cancer mouse model; Clinical trial in RA Ovarian cancer study	*In vitro* cellular models (non-ACS models) Level C	(Zhao et al., 2023; Alrafas et al., 2020; Khojah et al., 2018; Chen et al., 2022; Xing et al., 2022)
Fibrosis	Attenuates ECM deposition Blocks fibrotic progression	- Inhibits EMT (downregulates α-SMA, TGF-β1) - Inhibits TGF-β1-induced fibroblast activation - Antagonizes Hedgehog signaling - Downregulates AKT/foxo3a and eIF-2α/ATF4 pathways - Reduces collagen secretion	Lung degeneration model Wound healing model Renal fibrosis models	Animal models (non-ACS models) Level B	(Navarro et al., 2017; Bai et al., 2016; Song et al., 2024; Cui et al., 2022; Sun and Zheng, 2022; Zhang et al., 2016; Bai et al., 2014)

Abbreviations: AAA, Abdominal aortic aneurysm; ACS, Abdominal cocoon syndrome; AD, Alzheimer’s Disease; AKT, Protein Kinase B; AMD, Age-related Macular Degeneration; ATF4, Activating Transcription Factor 4; CAM, Chick chorioallantoic membrane; CD, Crohn’s Disease; COPD, Chronic obstructive pulmonary disease; CRP, C-Reactive Protein; CXCL-12, C-X-C Motif Chemokine Ligand 12; ECM, Extracellular matrix; eIF-2α, Eukaryotic Initiation Factor 2 alpha; EMT, Epithelial-Mesenchymal Transition; foxo3a, Forkhead Box Protein O3a; GSH, Glutathione; GSSG, Glutathione disulfide; HBMECs, Human Brain Microvascular Endothelial Cells; HO-1, Heme Oxygenase-1; HUVECs, Human Umbilical Vein Endothelial Cells; IBD, Inflammatory bowel disease; IFN-γ, Interferon-gamma; IL-10, Interleukin-10; IL-1β, Interleukin-1, beta; IL-6, Interleukin-6; IL-8, Interleukin-8; MAPK, Mitogen-Activated Protein Kinase; MDA, Malondialdehyde; MMP-9, Matrix Metalloproteinase-9; MMPs, Matrix Metalloproteinases; NF-κB, Nuclear Factor-kappa B; Nrf2, Nuclear factor erythroid 2–related factor 2; PCOS, Polycystic ovary syndrome; PGC-1α, Peroxisome Proliferator-Activated Receptor Gamma Coactivator 1-alpha; RA, Rheumatoid arthritis; ROS, Reactive oxygen species; SAA, Severe aplastic anemia; SIRT1, Sirtuin 1; SOD, Superoxide dismutase; TGF-β, Transforming Growth Factor-beta; Th17, T helper 17 cell; TNF-α, Tumor Necrosis Factor-alpha; Treg, Regulatory T cell; UC, Ulcerative colitis; VEGF, Vascular endothelial growth factor; α-SMA, Alpha-Smooth Muscle Actin.

Evidence strength grading (all evidence derived from non-ACS, models):

Level A: Consistent findings from ≥2 independent *in vivo* animal studies (non-ACS, fibrotic/inflammatory models).

Level B: single *in vivo* animal study, or consistent results from multiple *in vitro* cell experiments.

Level C: only one *in vitro* cell study; or inconsistent findings across independent studies.

**FIGURE 2:**
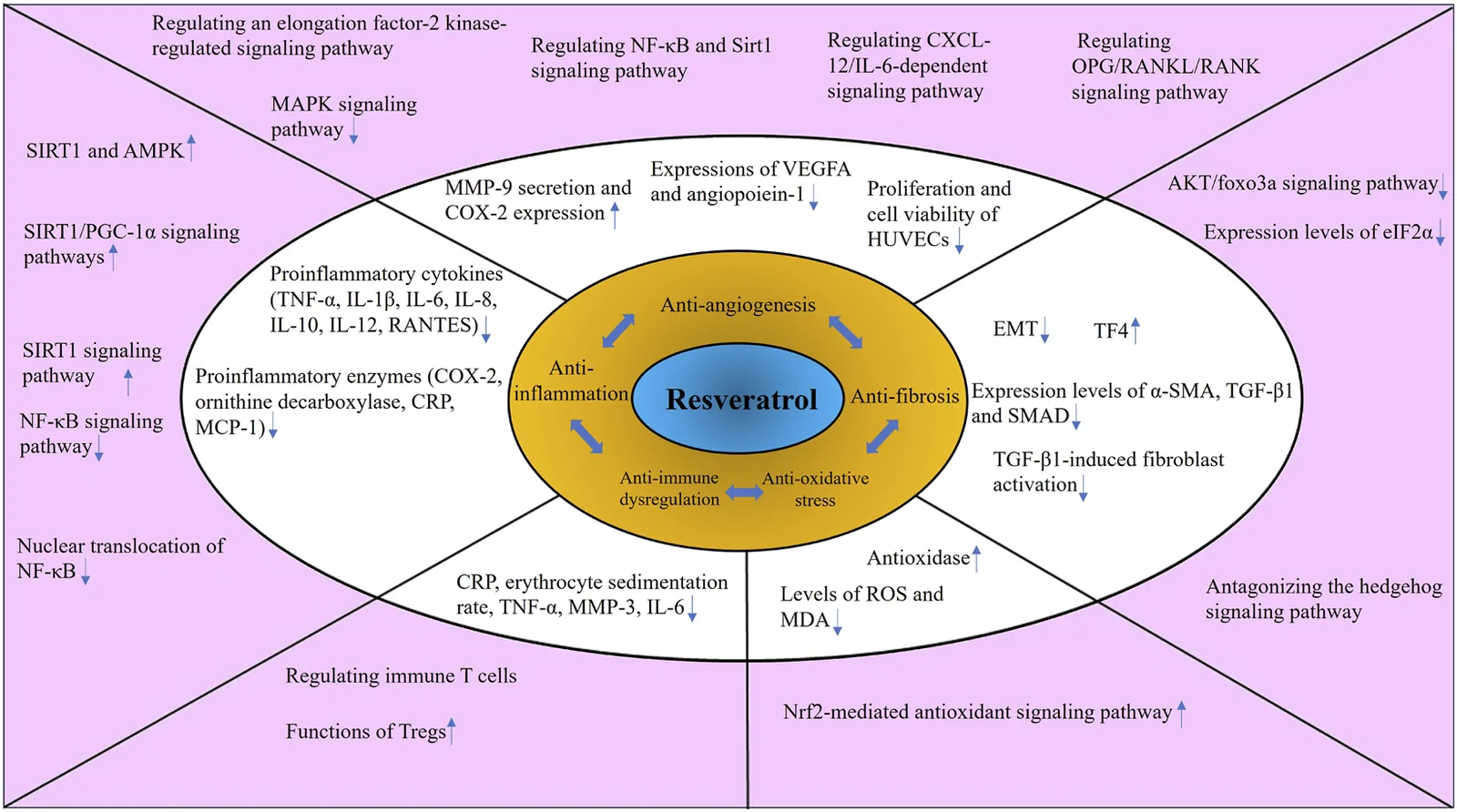
Beneficial effects of resveratrol on alleviating ACS and its possible underlying mechanisms.

### 4.1 Resveratrol’s protection against inflammation

Inflammation has been considered as one of the most important factors that cause ACS (Merkle and Wörnle, 2012). In most cases of ACS, chronic inflammation represents the common pathological features (Garosi et al., 2001). Resveratrol can alleviate various kinds of diseases by suppressing chronic inflammation.

In animal researches, reported that resveratrol can alleviate polycystic ovary syndrome (PCOS) in rats by inducing anti-inflammatory systems via SIRT1 and AMPK activation (Furat et al., 2018). Yang Z reviewed that resveratrol ameliorates abnormal follicular development by managing the level of chronic inflammation (Yang et al., 2020). Additionally, it also has been proved that resveratrol could alleviate bowel disorders such as IBD, colitis, and colon cancer by contributing an improvement in chronic inflammation. As for IBD, resveratrol exerted its protective role by reducing the expression levels of tumor necrosis factor-α (TNF-α), interleukin-8 (IL-8) and interferon-γ (IFN-γ). As for colitis, resveratrol exerted its protective role by the inhibition of pro-inflammatory factors (TNF-α as well as IL-1) and the increase of the anti-inflammatory cytokines such as IL-10. As for chronic colitis, oral administration of resveratrol could also have an ameliorated effect. Its inner mechanism was reducing local and systemic inflammatory cytokines, including TNF-α, IFN-γ, IL-1β, IL-6, IL-12, as well as RANTES. As for colon cancer, the durative resveratrol supplementation had been reported to suppress colon carcinogenesis by down-regulating the expressions of inflammatory proteins such as COX-2 and ornithine decarboxylase. also found that oral gavage of resveratrol could ameliorate intestinal permeability and inflammation through changing the gut microbiome in the diabetic mice model (Cai et al., 2020). Moreover, it also has been reported that resveratrol could alleviate respiratory disorders by contributing an improvement in chronic inflammation. For example, demonstrated that oral gavage of resveratrol could suppress the chronic inflammatory infiltration around the pulmonary arteries in the rat models (Xu et al., 2016). In smoking exposure rat and mice models, resveratrol can also exert its anti-inflammatory effects. Wang XL et al (2017) reported that resveratrol had a therapeutic effect in smoking induced chronic obstructive pulmonary disease (COPD) rat model, which is related to the inhibition of inflammatory response (decreased levels of IL-6 and IL-8). And its possible mechanism may be associated with the upregulation of the SIRT1/Peroxisome proliferator-activated receptor γ coactivator 1α (PGC-1α) signaling pathways. Wang et al. (2017) investigated that resveratrol could alleviate smoking-induced lung injury by inhibiting airway inflammation via suppressing the nuclear translocation of nuclear factor-κB (NF-κB) (Liu et al., 2014).

In clinical researches, resveratrol also has been reported to possess anti-inflammatory properties in endocrine system, respiratory system, skin, and nervous system. For example, oral administration of resveratrol could alleviate diabetes mellitus by contributing to the improvement of chronic inflammation (reduced levels of TNF-α, IL-1β, IL-6, as well as monocyte chemoattractant protein-1) (Mahjabeen et al., 2022), (Khodabandehloo et al., 2018). Otherwise, oral administration of resveratrol could alleviate COPD by decreasing levels of inflammatory markers such as IL-8 instead of C-reactive protein (CRP) (Beijers et al., 2020), (Culpitt et al., 2003). As for skin, wound, hypertrophic scar and skin photo-aging could be alleviated by resveratrol administration via inhibiting inflammation through upregulating SIRT1 signaling pathway and downregulating NF-κB signaling pathway (Hecker et al., 2022). Alzheimer’s disease (AD) is a common nervous disease, which manifests as the impairments of cognitive function and behavioral function. A retrospective study involving 119 AD patients performed by United States showed that oral administration of resveratrol for more than 52 weeks can alleviate AD via modulating neuro-inflammation (decreased levels of IL-12 and RANTES) (Moussa et al., 2017).

Taken together, both animal researches and clinical trials have supported that resveratrol could alleviate various types of diseases by inhibiting inflammation especially chronic inflammation. Although there are no studies have explored the direct association between resveratrol and ACS, we hypothesize that the anti-inflammatory effects of resveratrol will be beneficial on the treatment of ACS. As a pivotal role of ACS, the adverse effects induced by inflammation are not only inflammation itself, but also inflammation-associated pathological processes such as oxidative stress, as well as fibrosis, which will be thoroughly discussed in the following parts. Sustained peritoneal chronic inflammation acts as the initial trigger for fibrous membrane formation in ACS.

### 4.2 Resveratrol’s protection against oxidative stress

Oxidative stress is of great importance for the pathogenesis of ACS, and it also can deteriorate the prognosis of ACS. In most cases, oxidative stress is associated with inflammation, and it always induced by excessive inflammation. Therefore, attenuating oxidative stress is significant for alleviating ACS. As a type of TCM with anti-oxidative stress effects, resveratrol has been confirmed to alleviate various kinds of abdominal diseases such as abdominal aortic aneurysms (AAA), IBD, ulcerative colitis (UC), intestinal mucosal changes, Crohn’s disease (CD), as well as colon cancer by suppressing oxidative stress. Bilen A et al., Kaneko H et al., and Palmieri D et al., revealed that resveratrol was effective against development, rupture and lumen enlargement of AAA by reducing oxidative stress (Bilen et al., 2021; Kaneko et al., 2011; Palmieri et al., 2011). In addition, reviewed that resveratrol supplementation was a promising potential approach for the treatment of IBD due to its anti-oxidative stress effects (Hu Y. et al., 2019). Blagov AV et al., also reviewed that resveratrol could alleviate IBD via repairing the intestinal barrier and reducing its ROS level by inhibiting oxidative stress (Blagov et al., 2023). As for UC and CD, Samsamikor M et al., found that oral administration of resveratrol could improve the life quality of patients with UC partially through the inhibition of oxidative stress, which was manifested as decreased level of malondialdehyde (MDA), and increased level of superoxide dismutase (SOD) (Samsamikor et al., 2016). Otherwise, the therapeutic effects of resveratrol on CD are just like it on UC. As for intestinal mucosal changes, Volkan Temi et al., showed that resveratrol could attenuate the swell of intestinal villus and keep it as normal as possible under the pathological condition of obstructive jaundice. Its mechanism was that resveratrol could significantly decrease the level of ischemia-modified albumin, which was a potential mark of the degree of oxidative stress (Temi et al., 2014). In addition, it is also reported that resveratrol can treat colon cancer by inhibiting oxidative stress. ROS can induce excessive oxidative stress and contribute to the proliferation, as well as metastasis of colon cancer (Chiou et al., 2011). reported that resveratrol could prevent the development of colon cancer by inhibiting oxidative stress via activating NF-E2-related factor 2 (Nrf2)-mediated antioxidant signaling pathway (Zheng et al., 2019).

Taken together, we have summarized that resveratrol can alleviate abdominal diseases especially bowel disorders by inhibiting oxidative stress. As a type of rare bowel disorders, ACS is associated with some types of bowel pathological changes especially IBD and bowel tumors. Resveratrol has been confirmed to have a therapeutic effect on the treatment of these bowel disorders because of its anti-oxidative stress. Thus, we hypothesize that resveratrol can alleviate ACS through the anti-oxidative stress mechanism.

Furthermore, resveratrol has the poor solubility which limits its research value and clinical application. Besides, the poor solubility can also lead to the poor biological effects of resveratrol. To solve this problem, various strategies have been explored to overcome this limitation of resveratrol. For example, the combination of resveratrol and bioengineered materials is a promising thought. Zhu et al., reported that gelatin methacrylate, silk fibroin glycidyl methacrylate, mesoporous silica nanoparticles, as well as platelet-derived extracellular vesicles were great bioengineered materials, which are effective in releasing resveratrol (). Compared with resveratrol alone, the combinations of bioengineered materials with resveratrol are prone to achieve more efficient therapy and enhanced anti-oxidative stress effects. Kantayos et al., found that using yeast extracts (YEs) could enhance resveratrol production and antioxidant activity (Kantayos et al., 2022). And Eroğlu et al., showed that hyaluronic acid-dipalmitoyl phosphatidylcholine microparticles also could enhance the antioxidant activity of resveratrol, which was manifested as increased ratio of glutathione (GSH)/oxidized glutathione (GSSG) (Eroğlu et al., 2015). Thus, with the combinations of bioengineered materials, resveratrol can be more efficient in alleviating ACS by enhancing its antioxidant activity. Taken together, excess ROS within the peritoneal cavity of ACS aggravates mesothelial injury and accelerates peritoneal sclerosis.

### 4.3 Resveratrol’s protection against angiogenesis

As mentioned above, angiogenesis is closely associated with the pathogenesis of ACS and ignoring intervening with angiogenesis cannot be helpful to the treatment of ACS. It has been reported that resveratrol can regulate abnormal angiogenesis in various kinds of diseases. *In vitro*, A. Uvez et al., reported that resveratrol displayed an inhibitory effect on angiogenesis in that it could inhibit the proliferation and cell viability of human umbilical vein endothelial cells (HUVECs) (Uvez et al., 2020). Borhane Annab et al., reported that metalloproteinases (MMPs) and cyclooxygenase (COX)-2 were two important pro-angiogenic biomarkers. Resveratrol could efficiently increase the MMP-9 secretion and COX-2 expression of human brain microvascular endothelial cells (HBMECs) by regulating NF-κB and Sirt1 signaling pathway) (Annabi et al., 2012). Notably, in quiescent HBMECs, resveratrol moderately increases MMP-9 secretion and COX-2 expression to maintain physiological endothelial homeostasis. Under pathological stimuli such as inflammation and fibrosis, however, resveratrol suppresses the excessive upregulation of MMP-9 and COX-2, thereby inhibiting pathological angiogenesis. Rather than exerting universal inhibition, resveratrol modulates MMP-9 and COX-2 in a stress-dependent manner. In addition, Rajalaxmi Pradhan et al., reported that resveratrol nanoparticle could attenuate angiogenesis by the inhibition of cancer-associated fibroblasts (CAFs) in oral cancer by regulating Chemokine (C-X-C motif) ligand-12 (CXCL-12)/IL-6-dependent signaling pathway (Pradhan et al., 2023). Otherwise, Aslam A Khan et al., showed that resveratrol could modulate pathological angiogenesis of human microvascular endothelial cells (HMVECs) via regulating an elongation factor-2 kinase-regulated signaling pathway (Khan et al., 2010). Yang G et al., found that resveratrol could alleviate rheumatoid arthritis (RA) by suppressing angiogenesis through downregulating mitogen-activated protein kinase (MAPK) signaling pathway (Yang et al., 2018).

*In vivo,* A. Uvez et al., reported that resveratrol had the anti-angiogenic activity in a chick chorioallantois membrane (CAM) model. And compared with using resveratrol alone, the combination of resveratrol and doxorubicin could achieve a more satisfying results in the prevention and treatment of angiogenesis (Uvez et al., 2020). Xiong G et al., reported that resveratrol could inhibit angiogenesis of subchondral bone in osteoarthritis by regulating the osteoprotegerin (OPG)/RANK ligand (RANKL)/RANK signaling pathway and decreasing the expressions of vascular endothelial growth factor acceptor (VEGFA) and angiopoiein-1 (Xiong et al., 2021). Additionally, Aslam A Khan et al., demonstrated that resveratrol could inhibit the injury-induced angiogenesis of age-related macular degeneration (AMD) rat model by regulating the Sirt1-independent signaling pathway (Khan et al., 2010). Otherwise, resveratrol can inhibit angiogenesis, which has been thoroughly studied in anti-cancer researches. For example, found that the anti-angiogenic roles of resveratrol could provide the effective therapeutic strategy in colorectal cancer, of which the possible mechanism was suppressing the VEGF-mediated angiogenesis (Hu W. et al., 2019). Also, Varghese E et al., showed that resveratrol could regulate breast cancer progression by its anti-angiogenic effects. And its inner mechanism was the regulation of resveratrol of miRNA‐21, miRNA‐221/222, as well as miRNA‐27, which were prognostic biomarkers of the triple negative breast cancer (Varghese et al., 2020).

Pathological angiogenesis in ACS supplies cytokines and nutrients to sustain peritoneal fibroproliferation. Inhibiting abnormal angiogenesis cannot be ignored in the treatment of ACS and the aim of inhibiting abnormal angiogenesis is to prevent fibrosis. As mentioned above, resveratrol is able to inhibit abnormal angiogenesis so that there is hypothesized that resveratrol can suppress the fibrosis of ACS. However, more studies about the relationship between resveratrol and angiogenesis inhibition should be performed in the future.

### 4.4 Resveratrol’s protection against immune dysregulation

Immune dysregulation is commonly observed in ACS and its occurrence is associated with many factors such as inflammation, oxidative stress, as well as fibrosis, etc. The real relationship between immune dysregulation and ACS has not been deeply clarified. In most cases, immune dysregulation has been found to play an important role in the pathogenesis and development of ACS. However, in some cases, the development of ACS can also lead to the immune dysregulation, and the immune dysregulation will deteriorate ACS in turn.

Patients suffering with immune dysregulation are prone to infect with tuberculosis. It is well known that the abdominal fibrosis induced by intestinal tuberculosis and peritoneal tuberculosis is one of the causes of ACS. In addition, many diseases can cause ACS through immune dysregulation. For example, long-term peritoneal dialysis and its adjuvant drugs such as glucocorticosteroid can suppress the immune system of patients, which leads to the disorder of intestinal microenvironment, inducing intestinal fibrosis and ACS. Severe aplastic anemia (SAA) often occurs in immunocompromised patients and impairs intestinal barrier function as well as increased epithelial permeability, which will cause ACS (Zhao et al., 2023).

The immune regulating effects of resveratrol have been deeply investigated in many kinds of diseases. Alrafas, H.R et al., reported that resveratrol could be used to suppress colorectal cancer in a mouse model, leading to a deceleration of colorectal cancer development by regulating immune T cells (Alrafas et al., 2020). RA is a well-known autoimmune disease and it can be attenuated by resveratrol. A clinical trial has reported that resveratrol can greatly alleviate the disease status of RA patients and the laboratory results showed that some biochemical indicators reduced after the treatment of resveratrol, such as CRP, erythrocyte sedimentation rate, carbonylated osteocalcin, TNF-α, MMP-3, as well as IL-6 (Khojah et al., 2018). Moreover, Chen J et al., demonstrated resveratrol could inhibit the glycolysis in ovarian cancer cells so as to decrease the production of lactate, which weakens the suppressive function of T-regulatory cells (Tregs), and subsequently restores its anti-tumor immunity (Chen et al., 2022). In addition, as mentioned above, resveratrol can exert its biological properties with the promotion of the developed tissue bioengineered materials. Xing R et al., manifested that resveratrol could be encapsulated into quaternized chitosan-coated liposomes so as to forming a new type of nanoparticle delivery system. This new delivery system could make resveratrol delivery more effective, which enabled resveratrol-induced immune responses stronger and more durable (Xing et al., 2022). Therefore, local peritoneal immune imbalance forms a self-amplifying loop to maintain persistent fibrotic injury in ACS. The immune dysregulation effects of resveratrol may be a promising and potential target of treating ACS. And more basic researches and clinical trials should be performed to confirm it.

### 4.5 Resveratrol’s protection against fibrosis

Fibrosis is the most direct cause and final ending of ACS, controlling fibrosis is one of the most effective strategies to treat ACS. In most cases, fibrosis is the ultimate stage of many mechanisms interacting with each other in ACS. Fibrosis greatly contributes to the development and prognosis of ACS, of which the possible mechanisms include parenchymal cell reduction, fibroblast activation, epithelial-mesenchymal transition (EMT), as well as collagen protein secretion and deposition, etc. In the aging lung degeneration mice model, Sonia Navarro et al., reported that resveratrol treatments could block the parenchymal cell DNA damage and maintain the lung structure (Navarro et al., 2017). Bai XZ et al., showed that resveratrol treatment could lead to the organized and orderly collagen fiber in the wound healing mice model, of which the possible mechanism was to downregulate SIRT1 expression so as to inhibit tissue growth factor-β1 (TGF-β1)-induced fibroblast activation (Bai et al., 2016). As for EMT, Song L et al., found that resveratrol can downregulate α-smooth muscle actin (α-SMA), TGF-β1, and specific E3 ubiquitin ligase 2 (SMAD) expression levels, which led to the inhibition of EMT (Song et al., 2024). Notably, this regulatory pattern is context-dependent. In inflammatory immune cells (e.g., macrophages), resveratrol upregulates SIRT1 and suppresses inflammatory cascades via NF-κB deacetylation. In TGF-β1-activated fibroblasts, however, profibrotic signaling rewires the cellular transcriptional landscape. Under this profibrotic priming condition, resveratrol reduces SIRT1 levels and thereby inhibits fibroblast-to-myofibroblast transition. In addition, it also has been reported that resveratrol can exert its protective effect against UVB-induced aging *in vitro* and vivo by reducing the secretion of collagen protein (Cui et al., 2022). Otherwise, several in-depth researches have investigated the molecular mechanism of the inhibiting effects of resveratrol on fibrosis. Sun et al., supported that resveratrol can inhibit renal interstitial fibrosis by downregulating protein kinase B (serine/threonine kinase, AKT)/forkhead box protein O3a (foxo3a) signaling pathway (Sun and Zheng, 2022). Zhang et al., also supported that resveratrol could inhibit renal interstitial fibrosis by downregulating the expression levels of eukaryotic initiation factor-2α (eIF-2α) and activating transcription factor 4 (TF4) (Zhang et al., 2016). Moreover, Bai et al., supported that resveratrol could inhibit EMT and renal fibrosis via antagonizing the hedgehog signaling pathway (Bai et al., 2014).

Finally, peritoneal fibrosis produces the fibrous cocoon, which mechanically entraps intestines and causes bowel obstruction in ACS. Through the summary of related researches and reviews in this part, we find that researches investigating the relationships between resveratrol and fibrosis in ACS are few. However, investigating how to relieve fibrosis is of a great significance for treating ACS so that these types of original researches have a promising prospective. Therefore, to clarify its underlying mechanisms deeply and clearly, more and more relevant researches should be performed in the next step.

## 5 Pharmacokinetic limitations and optimized delivery strategies of resveratrol for ACS treatment

Although resveratrol demonstrates significant anti-inflammatory, antioxidant, and anti-fibrotic potential in the treatment of ACS, its clinical application still faces major challenges. These challenges primarily stem from resveratrol’s inherent pharmacokinetic limitations, including physicochemical instability and low bioavailability. A thorough understanding of these restrictive factors and the development of corresponding optimization strategies are crucial for realizing resveratrol’s therapeutic value in fibrotic diseases such as ACS (Table 3).

**TABLE 3:** Major physicochemical limitations of resveratrol and corresponding advanced delivery strategies.

Category	Specific limitations	Impact on therapy	Optimization strategy	Mechanism of action	Key outcomes	Technological maturity	References
Solubility & Dissolution	Low water solubility due to hydrophobic structure	Low dissolution rate and absorption efficiency; incomplete oral bioavailability	Nanocarrier Systems • Mesoporous silica nanoparticles (MSNs) • Polymeric micelles (e.g., PLGA-PEG)	• High surface area and pore volume for encapsulation • Core-shell structure solubilizes resveratrol in hydrophobic core	• Drug loading efficiency up to 90% (MSNs) • Enhanced solubility and stability	Laboratory stage	(Bohara et al., 2022; Ali et al., 2024; Harwansh et al., 2023)
Stability & Metabolism	Rapid isomerization (trans- to cis-); Extensive pre-systemic metabolism (UGTs, SULTs, CYP450)	Short half-life (4–6 h in GI fluid); Low oral bioavailability (<1%); Loss of bioactivity	Structural Modification & Prodrugs • Acetylation (e.g., 3,5-diacetyl-resveratrol) • PEGylation • Liver-targeting prodrugs	• Blocking metabolic sites (e.g., hydroxyl groups) • Targeting moieties enhance organ-specific uptake	• Plasma half-life extended from 8 min to 120 min • Hepatic uptake increased to >70%	Laboratory stage	(Yakubu and Pandey, 2024); (Mora-Gutierrez et al., 2020)
Narrow Therapeutic Window	Biphasic, concentration-dependent effect (Anti-oxidant at <10 μM, pro-oxidant at >100 μM)	Risk of paradoxical effects; Unsuitable for long-term chronic use	Stimuli-Responsive Nanosystems • Redox-sensitive nanoparticles • pH-responsive carriers (e.g., ZIF-8 MOFs)	• Drug release triggered by high ROS in diseased microenvironment • Degradation in weakly acidic sites	• 3–5 times greater drug release at target sites • Enhanced targeting precision	Laboratory stage (not in ACS models)	(Sarfraz et al., 2023; Ma et al., 2025; Li L. et al., 2023; Hao et al., 2022)
Systemic Absorption & Distribution	Extensive first-pass metabolism; Poor distribution to fibrotic sites in ACS	Failure to achieve therapeutic plasma concentration; Physical barrier of abdominal adhesions	Alternative Administration Routes • Intraperitoneal delivery (e.g., Thermosensitive *in situ* gels) • Transdermal delivery (Dissolvable microneedle arrays)	• Local delivery bypasses first-pass metabolism • Sustained release at the disease site	• Targeted therapy for advanced HCC with ascites • Sustained drug release	Laboratory stage	(Wen et al., 2020); (Li et al., 2024)

Abbreviations: ACS, Abdominal cocoon syndrome; CYP450, Cytochrome P450 enzyme system; GI, Gastrointestinal; HCC, Hepatocellular carcinoma; MOFs, Metal-Organic Frameworks; PEG, Polyethylene glycol; PLGA, Poly (lactic-co-glycolic acid); ROS, Reactive oxygen species; SULTs, Sulfotransferases; UGTs, UDP-glucuronosyltransferases.

### 5.1 Physicochemical limitations of resveratrol and their impacts on clinical applications

Resveratrol, as a natural polyphenolic compound with significant pharmacological activity, demonstrates multiple therapeutic advantages in the treatment of ACS. However, its clinical application is systematically constrained by a series of interrelated physicochemical limitations that form a complex pharmacokinetic barrier network (Ben Dassi et al., 2024). From a molecular structural perspective, the hydrophobic aromatic ring structure of resveratrol determines its extremely low water solubility. This characteristic not only affects its formulation development but also significantly reduces its dissolution rate and absorption efficiency in the gastrointestinal tract (Ren et al., 2021). More critically, resveratrol undergoes complex metabolic transformations *in vivo*: in intestinal epithelial cells, its hydroxyl groups are readily catalyzed by enzymes such as UGT1A1 and SULT1A1 to undergo glucuronidation and sulfation; after entering the portal venous circulation, it is further extensively metabolized by hepatic CYP450 enzyme systems (particularly CYP1A2 and CYP3A4) (Yang et al., 2025). This multi-level metabolic conversion results in an oral bioavailability typically below 1%, with significant interindividual variability.

From the perspective of drug stability, resveratrol is found to exist in two isomeric forms: *cis-* and *trans-*, which readily interconverts under conditions of light exposure, heating, or pH variation (Leischner et al., 2021). Experimental data demonstrated that in simulated gastrointestinal fluids, the half-life of the trans-configuration was only 4–6 h, while bioactivity studies revealed that the antioxidant activity of the cis-isomer was merely 30%–50% of that of the trans-form (Jaa et al., 2025), (Chung and Hyun, 2021). This chemical instability poses significant challenges for pharmaceutical processing and quality control. From a pharmacodynamic perspective, resveratrol exhibits a characteristic concentration-dependent biphasic effect: at low concentrations (<10 μM), it demonstrated antioxidant activity, whereas with increasing dose levels, its function shifted from antioxidant to pro-oxidant. When resveratrol reached 100 μM, treated groups showed 5–13 folds mitochondrial metabolic alterations compared to controls, providing direct evidence for its high-concentration oxidative stress induction mechanism (Falcone and Rushing, 2025). This narrow therapeutic window poses particular challenges in chronic conditions like ACS requiring long-term administration, as interpatient variability in absorption and metabolism could lead to plasma concentration fluctuations beyond the safe range, potentially resulting in paradoxical therapeutic outcomes (Varga et al., 2024).

The limited absorbed quantity then faces extensive first-pass metabolism, while accumulated metabolites may further inhibit subsequent metabolism of the parent compound through feedback mechanisms (Ben Dassi et al., 2024). This complex pharmacokinetic profile makes it particularly challenging to establish stable and effective plasma concentration-time curves for resveratrol in ACS treatment, significantly constraining its clinical translation potential. Notably, these limitations may be further exacerbated in the unique pathological microenvironment of ACS: fibrotic alterations in peritoneal vasculature can impair drug distribution, chronic inflammatory states may modify metabolic enzyme activity, and physical barriers formed by abdominal adhesions can obstruct local drug delivery (Goh et al., 2017). Collectively, these factors constitute multiple obstacles that must be overcome in resveratrol-based ACS therapy, while simultaneously highlighting the critical need for developing novel drug delivery systems specifically designed to address these challenges.

### 5.2 Strategies for optimizing resveratrol delivery systems

To address the significant pharmacokinetic limitations of resveratrol, researchers have conducted systematic optimization studies in recent years from three dimensions: nanotechnology, structural modification, and novel drug delivery routes. These delivery strategies cannot be evaluated in isolation. Their practical utility must be assessed against ACS specific pathological constraints, including the dense fibrotic physical barrier of the peritoneal cocoon, ROS rich inflamed peritoneal microenvironment, and altered drug metabolic profiles in high-risk ACS populations such as long-term peritoneal dialysis patients. In terms of nanocarrier technology, beyond traditional liposomal systems, novel nanostructures such as mesoporous silica nanoparticles achieve drug loading efficiencies as high as 90% due to their well-ordered pore structures (pore size: 2–10 nm). Furthermore, surface modification with targeting molecules such as RGD peptides enhances their specific accumulation in fibrotic tissues (Bohara et al., 2022). For ACS, such nanoparticles must penetrate the dense fibrous peritoneal membrane to reach activated mesothelial cells and fibroblasts; most current mesoporous silica carrier evidence remains preclinical, and their penetration capacity across thick peritoneal fibrotic tissue has not been validated in ACS animal models. Meanwhile, polymeric micelle systems based on amphiphilic block copolymers (e.g., PLGA-PEG) effectively address resveratrol’s solubility issues through their core-shell structure, with critical micelle concentrations as low as 0.001 mM, demonstrating excellent stability under physiological conditions (Ali et al., 2024), (Harwansh et al., 2023). PLGA-PEG micelles show promise for peritoneal administration in ACS, yet risks of micelle entrapment within fibrous adhesions and off target uptake by inflammatory immune cells require further *in vivo* verification. Recent studies have also revealed that combining resveratrol with metal-organic frameworks such as ZIF-8 not only significantly increases drug loading capacity but also enables pH-responsive release by leveraging the weakly acidic tumor microenvironment. This strategy exhibits unique advantages in treating peritoneal fibrosis (Sarfraz et al., 2023). This pH responsive property is potentially adaptable to the acidic inflamed peritoneal microenvironment in ACS, though all ZIF-8 based resveratrol formulations remain in early laboratory exploration without peritoneal fibrosis related *in vivo* data.

Of particular significance is the development of intelligent stimulus-responsive delivery systems, where redox-sensitive nanoparticles utilize the highly expressed ROS in fibrotic peritoneal microenvironments to trigger drug release. By incorporating responsive linkers such as thioether or selenium bonds, these systems achieve 3–5 times greater drug release at lesion sites compared to normal tissues (Ma et al., 2025), (Li L. et al., 2023). This ROS triggers release matches the oxidative stress signature of ACS peritoneal lesions and can reduce off target drug exposure. However, excessive ROS scavenging may interfere with normal cellular redox homeostasis, and this strategy is still confined to cell and small animal experiments. Recent advances demonstrate that combining ROS responsive and enzyme responsive dual trigger mechanisms further enhances targeting precision (Hao et al., 2022). Regarding structural modification strategies, beyond conventional methylation and esterification approaches, prodrug design has achieved critical breakthroughs: liver-targeting prodrugs constructed by linking resveratrol to targeting moieties through enzyme-cleavable bonds not only increase hepatic uptake to over 70% but also enable controlled release under hepatic esterase activity, while 3,5-diacetylated resveratrol developed via metabolic site-blocking strategy significantly extends plasma half-life from 8 min to 120 min (Yakubu and Pandey, 2024). Systemic resveratrol prodrugs improve oral bioavailability by mitigating hepatic first pass metabolism, but they cannot overcome the fibrous peritoneal barrier in ACS. High systemic drug exposure also raises pro-oxidant risk for peritoneal dialysis patients with impaired metabolism. Recent studies further reveal that PEGylated resveratrol derivatives markedly improve pharmacokinetic properties while maintaining pharmacological activity (Mora-Gutierrez et al., 2020).

In terms of optimized administration routes, a clear distinction between systemic oral delivery and local intraperitoneal delivery is required for ACS application. Oral administration suffers from inherent drawbacks: even modified prodrugs cannot bypass the thick fibrous peritoneal barrier, and fluctuating systemic drug concentrations may fail to reach therapeutic thresholds within the encapsulated peritoneal cavity. Localized intraperitoneal delivery systems demonstrate unique advantages, particularly thermosensitive *in situ* gel formulations, which can directly retain resveratrol within the peritoneal cavity, bypass gastrointestinal and hepatic first pass metabolism, and act locally on the fibrotic peritoneal membrane. Studies show that co-loading resveratrol microspheres with cisplatin in Pluronic F127 thermosensitive hydrogels for intraperitoneal administration enables targeted therapy for advanced hepatocellular carcinoma with ascites (Wen et al., 2020). For ACS, thermosensitive hydrogels must sustain drug release while resisting rapid clearance by peritoneal fluid and avoiding entrapment inside dense fibrous cocoons. This application scenario remains untested and stays at the preclinical concept stage. Recent research has further explored microneedle array-based transdermal delivery systems, where resveratrol nanocrystals loaded into dissolvable microneedles effectively bypass first pass metabolism while achieving sustained drug release (Li et al., 2024). Transdermal microneedles cannot achieve direct peritoneal targeting and are less suitable for focal peritoneal fibrosis in ACS. The synergistic application of these innovative delivery strategies, such as combining nanoparticle drug carriers with local gel delivery systems, shows promise in overcoming multiple barriers to clinical translation of resveratrol. Particularly noteworthy is the emerging field of artificial intelligence-assisted delivery system design, where machine learning algorithms optimize carrier material selection and formulation development, significantly improving research efficiency. These technological advances not only open new avenues for resveratrol application in treating fibrotic diseases like ACS, but also provide valuable references for developing other poorly soluble drugs. However, most of the above delivery strategies have not been validated in abdominal cocoon syndrome or peritoneal fibrosis models. For patients with ACS, intraperitoneal injection faces limitations including poor tissue penetration, insufficient local retention, and safety risks such as secondary infection and aggravated peritoneal adhesion. In addition, transdermal microneedles are unsuitable for local targeted drug delivery in ACS, as they cannot penetrate the thick fibrous cocoon to deliver drugs into the encapsulated peritoneal cavity. In summary, although various delivery technologies generally improve the solubility and stability of resveratrol, their adaptability, biosafety and peritoneal pharmacokinetic properties under the unique pathological conditions of ACS remain unproven and require rigorous *in vivo* verification.

## 6 Research gaps, key scientific challenges and quality-related considerations of resveratrol for ACS

Current pharmacological research on ACS remains extremely insufficient. Limited clinical cases and insufficient basic studies greatly restrict the development of effective therapeutic strategies for ACS. At present, standardized and targeted drug intervention systems specifically designed for ACS are still absent. In terms of resveratrol research, relevant studies mainly focus on common fibrotic diseases, while systematic investigations regarding peritoneal fibrosis and ACS are severely lacking. No direct evidence has confirmed the definite efficacy, optimal dosage and exact mechanism of resveratrol in ACS treatment. The application value of resveratrol in improving the peritoneal microenvironment and inhibiting ACS progression still needs further verification. Several critical scientific problems urgently need to be solved in future studies. Stable and standardized ACS animal models should be established to support basic pharmacological exploration. The local intraperitoneal pharmacokinetic characteristics of resveratrol require systematic clarification. In addition, drug penetration efficiency in fibrotic peritoneal lesions and the drug response characteristics of ACS pathological microenvironment are key research directions to promote the clinical transformation of resveratrol.

As a natural bioactive compound, resveratrol exhibits significant variations in content across different botanical sources. Common resveratrol-rich materials include grapes, red wine, peanuts, berries, and *Polygonum cuspidatum*. The accumulation level of resveratrol differs substantially among these species, resulting in inconsistent raw material activity and pharmacological potency. Such botanical heterogeneity emphasizes the necessity of standardized source screening for experimental and translational studies. Resveratrol exists mainly in trans and cis isomeric forms, which differ greatly in chemical stability and biological activity. Trans-resveratrol is the predominant and more stable form in nature with stronger antioxidant and anti-fibrotic activity. In contrast, cis-resveratrol presents poorer structural stability and lower pharmacological efficacy. Environmental conditions including light exposure, ultraviolet radiation and pH changes can trigger mutual isomerization, further altering the effective activity of resveratrol preparations. Extraction and purification techniques significantly affect the final structural integrity, purity and bioactivity of resveratrol. Different extraction methods, solvent systems and purification procedures may lead to residual impurities, structural transformation and varied component ratios. Optimized processing protocols are essential to preserve isomer stability and guarantee batch-to-batch consistency. Rigorous quantitative detection, impurity exclusion and isomer identification are required to ensure experimental repeatability and pharmacological reliability. Uniform quality specifications help avoid result deviation caused by raw material differences and improve the scientific rigor of preclinical and translational studies.

Resveratrol exhibits typical biphasic dose-dependent bioactivities. At low and moderate concentrations, it mainly exerts antioxidant, anti-inflammatory and anti-fibrotic effects, whereas high concentrations may induce pro-oxidant responses, trigger excessive reactive oxygen species accumulation and lead to cellular oxidative damage. Long-term high-dose resveratrol supplementation can alter hepatic enzyme activity and disrupt normal hepatic metabolism. Moreover, resveratrol inhibits cytochrome P450 isoforms, which may cause drug-drug interactions when combined with other agents and compromise drug metabolism and safety. Notably, there is a marked gap between the safe daily intake for humans and pharmacologically effective doses. The European Food Safety Authority sets the safe daily intake limit of resveratrol at ≤150 mg for adults. Nevertheless, most preclinical studies achieving anti-fibrotic and anti-angiogenic effects adopt concentrations far exceeding this human safe threshold. Such dose discrepancy represents a major barrier to the clinical translation of resveratrol as an adjuvant therapy for abdominal cocoon syndrome. Collectively, although resveratrol may serve as a potential candidate for abdominal cocoon syndrome intervention, its biphasic dose effects, poorly defined long-term toxicity, potential drug interaction risks and substantial mismatch between preclinical effective doses and human safety limits greatly restrict its clinical utility. Further dose-finding experiments, toxicological validation and pharmacological investigations are required for optimization.

## 7 Conclusions and prospects

Resveratrol exhibits multi-target and multi-pathway therapeutic potential for ACS. It should be highlighted that all therapeutic effects proposed in this review are hypothetical and indirectly inferred from findings of other fibrotic disease models. Direct cellular, animal or clinical evidence supporting resveratrol intervention against ACS is currently lacking. This review systematically summarizes the putative interventional effects of resveratrol on pathological processes related to ACS, including inflammation, oxidative stress, pathological angiogenesis, immune dysregulation and fibrosis. Resveratrol can simultaneously modulate multiple core pathological links of ACS. It suppresses chronic inflammation via the NF-κB and TGF-β/Smad pathways, alleviates oxidative stress by activating the Nrf2/HO-1 antioxidant pathway, inhibits pathological angiogenesis, restores immune homeostasis through balancing Th17/Treg subsets, and delays fibrotic progression by blocking epithelial-mesenchymal transition and excessive collagen deposition. These pharmacological pathways cooperate synergistically to form a multi-dimensional regulatory network. Nevertheless, the clinical translation of resveratrol for ACS treatment is limited by its intrinsic pharmacokinetic drawbacks and narrow therapeutic window. The clinical translation of resveratrol for ACS is also limited by several practical constraints. The rarity of ACS hinders model establishment and clinical research. Its biphasic, dose dependent effect (antioxidant at low doses, pro-oxidant at high doses) poses safety risks for intraperitoneal delivery. Most nano delivery systems are far from clinical application. Moreover, the distinctions among dietary resveratrol, supplements and nanomedicines should be clarified, since normal dietary intake cannot reach pharmacologically effective concentrations.

With advances in nanomedicine and precision medicine, resveratrol may represent a promising adjunctive therapy for ACS. Successful translation demands interdisciplinary collaboration of pharmaceutical, clinical and food-related researchers to connect basic pharmacological findings with clinical practice.
